# Deep learning segmentation of paediatric brain tumours using diffusion-weighted MRI: towards an early, *in vivo* classification pipeline

**DOI:** 10.1093/braincomms/fcag317

**Published:** 2026-08-17

**Authors:** Daniel J Griffiths-King, Timothy Mulvany, Heather E L Rose, Andrew C Peet, John R Apps, Theodoros N Arvanitis, Jan Novak

**Affiliations:** Aston Institute of Health and Neurodevelopment, College of Health and Life Sciences, Aston University, Birmingham B4 7TE, UK; Aston Institute of Health and Neurodevelopment, College of Health and Life Sciences, Aston University, Birmingham B4 7TE, UK; Medical Physics and Clinical Engineering, Nottingham University Hospitals NHS Trust, Nottingham NG5 1PB, UK; College of Engineering and Physical Sci., Aston University, Birmingham B4 7TE, UK; Department of Oncology, Birmingham Children’s Hospital, Birmingham B4 6NH, UK; Department of Cancer and Genomic Sciences, University of Birmingham, Birmingham B15 2TT, UK; Department of Oncology, Birmingham Children’s Hospital, Birmingham B4 6NH, UK; Department of Cancer and Genomic Sciences, University of Birmingham, Birmingham B15 2TT, UK; School of Engineering, University of Birmingham, Birmingham B15 2TT, UK; Aston Institute of Health and Neurodevelopment, College of Health and Life Sciences, Aston University, Birmingham B4 7TE, UK

**Keywords:** paediatric, brain tumour, deep learning, diffusion MRI, automatic segmentation

## Abstract

Diffusion-weighted MRI (DWI) can offer vital quantitative biomarkers to understand paediatric brain tumours to benefit clinical management. However, extraction of these markers requires delineation of the tumour margins, which is time-consuming, requires expertise and can still be highly variable. Automated approaches using deep learning to generate these tumour regions exist, but to our knowledge, few exist using only DWI as an input modality, with none in paediatrics. This retrospective study develops an automated segmentation approach, leveraging transfer learning and multi-modal ensembling to tackle the issues specific to this DWI-only approach. Using the Imaging of Tumors study data, we analysed data from 107 paediatric brain tumour patients. Using a 3D convolutional neural network, namely DeepMedic, to perform automatic segmentations, we demonstrate the benefit of these approaches for this task. We assess the accuracy of these predicted segmentations in comparison to the ‘ground truth’ manual annotations, with a median Dice score of 0.63 achieved by the best-performing model. The current study highlights the potential of this approach to be implemented in future clinical decision support tools using DWI, but more work is needed to improve segmentation accuracy or establish current performance as ‘sufficient’ for the purposes that these segmentations are required.

## Teaser

Griffiths-King *et al*. proposed a convolutional neural network (CNN) approach for automated segmentation of paediatric brain tumours, exclusively from diffusion-weighted MRI (DWI). Through pre-training on T2-weighted MRI from open-access paediatric data, then leveraging transfer learning, the model achieves a Dice score of 0.63. Findings demonstrate the feasibility of automated segmentation for native DWI biomarker extraction.

## Graphical Abstract

**Graphical Abstract:**
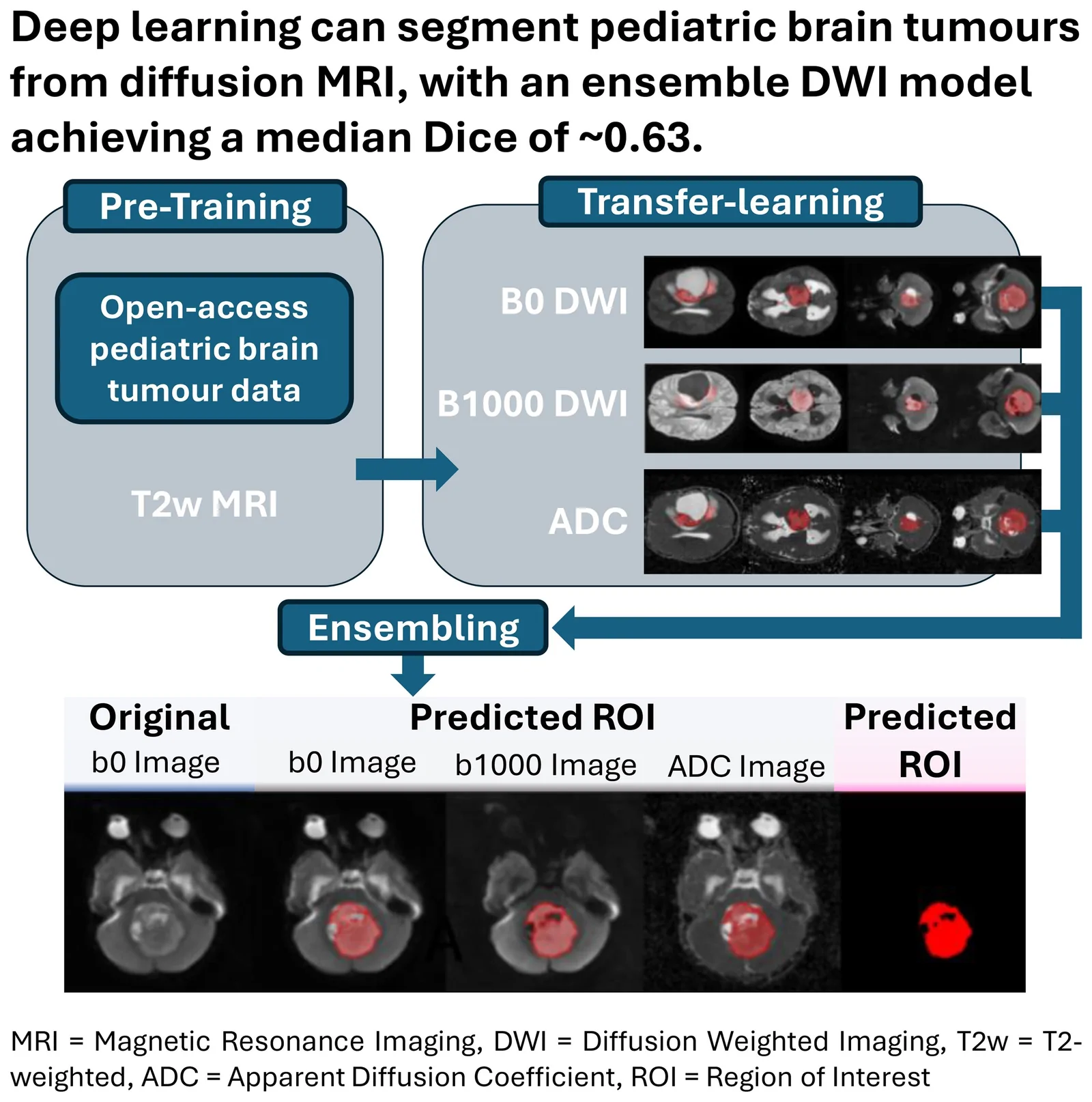

## Introduction

Diffusion-weighted MRI (DWI) is a standard imaging modality for neuroradiological assessment of paediatric brain tumours. DWI is recommended by the European Society for Paediatric Oncology (SIOPE) and the Response Assessment in Pediatric Neuro-Oncology (RAPNO) working group MRI guidelines for imaging patients with central nervous system tumours.^1,2^ DWI generates signal contrast based on diffusion of water molecules. Quantitative biomarkers that can be numerically derived from this diffusion-weighted signal, such as the apparent diffusion coefficient (ADC), provide insights into the tumour microenvironment (e.g. cellularity of tumour tissue^3,4^) and have been used to differentiate paediatric brain tumours *in vivo* based on diagnostic classification,^3,5-7^ tumour grade^8,9^ and (pseudo)progression.^10^

Regions of interest (ROIs), manual annotations drawn around visible tumour margins, are required to extract DWI metrics (such as ADC) from within tumour tissue. These metrics can be subsequently incorporated into downstream analyses, such as diagnostic classifiers, as biomarker candidates. Manual delineation of these ROIs can be conducted in several ways. Most simply, geometric masks (such as elliptical ROIs of a given size) can be placed within the boundaries of the pathological tissue. However, previous research has highlighted poor interobserver reliability of these approaches in paediatric and adult brain tumours,^11,12^ with significant differences in ADC values recovered from ROIs placed by different radiologists. This is potentially due to both heterogeneity within the tumour itself and subjective placement of these ROIs.

Whole-tumour ROI approaches may mitigate these issues, requiring individuals to manually delineate the tissue on the MRI deemed to be pathology, according to given *a priori* ‘rules’. Individuals with the requisite neuroradiological expertise to draw these whole-tumour ROIs accurately have limited capacity to conduct this time-consuming process in the context of existing clinical workload. Additionally, even expert-drawn ROIs are subject to inter-/intra-rater variability.^13-15^

Typically, ADC maps have been shown to have good reproducibility across different scanners of varying field strengths and acquisition parameters,^16^ but this variability in manually drawn/placed ROIs demonstrates the negative impact of manually drawn ROIs on the reproducibility of these metrics. Therefore, to support DWI biomarker extraction and development in paediatric brain tumours, automated segmentation approaches for the generation of tumour ROIs are highly appealing; they reduce the labour required and support consistency in drawn ROIs within/between cases by removing inter-rater effects.

Automatic segmentation of paediatric brain tumours, leveraging recent developments in deep learning models, has been relatively successful in recent years.^17^ Much of the work segmenting paediatric brain tumours (e.g. ^18,19^) uses the open-access, multi-consortium dataset of paediatric high-grade glioma from the Brain Tumor Segmentation in Pediatrics (BraTS-PED) 2023/24 challenges,^20-22^ with relatively high accuracy compared to manually delineated ROIs as the ‘ground truth’ segmentation.

However, current automated approaches do not typically segment pathology on the DWI image; instead, they use using multiparametric MRI (mpMRI) as inputs to segmentation algorithms. These integrate MRI modalities/contrasts that are acquired as part of clinical care as the ‘standard’ structural MR protocol for this population [T1-weighted MRI (T1w), contrast-enhanced T1w MRI using a contrast agent (T1w-CE), T2-weighted MRI (T2w), fluid-attenuated inversion recovery MRI (FLAIR), as per SIOPE/RAPNO guidelines^1,2^]. This mpMRI approach, excluding DWI, poses multiple additional challenges for the purpose of generating ROIs to extract DWI metrics. It requires co-registration between DWI and mpMRI across modalities, which can be challenging in the presence of gross pathology/abnormality and geometric distortions common to DWI acquisitions,^23^ with error propagation due to mis-registration. Furthermore, an mpMRI model for tumour segmentation requires additional computational burden, including preprocessing and quality assurance checks. Also, utilizing multiple input MRI modalities subsequently presents multiple additional opportunities for missing or poor data quality at the individual case level. That is to say, for any new cases, any one of the four input modalities needs to be missing or of poor quality to prevent inference on said case.

To our knowledge, no study has used deep learning to segment paediatric brain tumours exclusively from DWI, though studies have investigated other tumour types. A previous study demonstrated the utility of such an approach in paediatric osteosarcoma.^24^ Deep neural network approaches were able to segment osteosarcoma tumours from DWI with relatively high accuracy. Further studies have used DWI-only automated segmentation for pelvic lymph nodes,^25^ extrahepatic cholangiocarcinoma,^26^ cervical cancer^27^ and rectal tumours^28^ or used only ADC maps for segmenting prostate cancer.^29^ In a study of automated segmentation of bladder cancer, models using maps of ADC values, extracted from DWI, outperformed other single-modality models (e.g. T1w or T2w MRI).^30^ A similar deep learning study for segmenting pelvic and sacral tumours from MRI tested multiple combinations of MRI sequences (as a means of testing input-level fusion) and found that DWI commonly appeared in the best-performing models.^31^ Additional studies have used semi-automated and feature engineering approaches (such as thresholding) to segment multiple types of brain lesions, including solid tumours, from DWI.^32-34^ These studies did not establish generalizability through cross-validation (CV) frameworks.

The current study proposes a framework using a 3D, multi-scale CNN for the automated segmentation of paediatric brain tumours, solely using DWI as an input, for the purpose of extracting quantitative biomarkers. The specific goal of such a pipeline is to implement these automatic segmentations/ROIs into a diagnostic classifier, using DWI imaging biomarkers to predict tumour diagnosis *in vivo*.

We employ an existing CNN tool called DeepMedic.^35^ Details of the DeepMedic architecture can be found elsewhere, but briefly, this multi-scale 3D CNN is a specialized tool for segmenting brain lesions in medical images and has performed successfully in several artificial intelligence (AI) ‘challenge’ scenarios (i.e.^36^). The DeepMedic architecture utilizes dual pathways, one each for normal- and low-resolution sampling, enabling the simultaneous processing of both local and global context, which is then combined in the final densely connected layers. It uses patch-based processing for efficient memory usage, along with balanced training across both lesion-centred and background-centred patches, to deal with issues of class imbalance.

Due to the rarity of this disease,^37^ in this population, there is limited availability of open-access MRI datasets and high-quality annotations (other than the aforementioned BraTS-PEDs datasets^20,21^). Typically, there is insufficient data to train the large number of parameters required by deep-learning models, especially for the modality of DWI. Therefore, successfully training deep-learning models can be challenging.

A potential solution to this is transfer learning, exploiting previously learnt relationships between input and output data from a similar deep-learning task. Previous paediatric studies have achieved this by pre-training algorithms on adult MRI data. One study segmenting paediatric low-grade glioma from T2w MRI found that pre-training on adult data significantly outperformed (i) applying an adult model directly to paediatric data or (ii) training a model from scratch on the paediatric data.^38^ Whilst training on sufficient paediatric data may eventually remove the need for transfer learning,^38^ it remains a viable solution into improving model training.

In the current study, we specifically conducted inductive transfer learning^39^ to account for the rarity of paediatric brain tumours and the relatively low abundance of DWI data, both of which can harm sample sizes and have the ability to train robust learners. DWI produces multiple constituent imaging sets, including those with b0 and b1000 weighting, and subsequent calculations can generate spatial maps of ADC values. These three pre-processed image types (b0, b1000, ADC) are used as inputs for the current models in this study. This enables us to generate multiple input images from a single MRI modality, similar to the approach in.^27^ Our tumour segmentation model was initially pre-trained on an existing, open-access MRI dataset—from the 2023 BraTS-PEDs challenge,^21^ before being retrained and applied on a local dataset comprising only DWI. To enable transfer learning whilst minimizing domain shift, the model was trained using only T2w MRI as the input modality, selected because the image contrast is closest to the expected b0 contrast. Whilst the type of population, source and target tasks remain highly similar, the target domain for the new model was shifted in terms of modality, DWI instead of T2w MRI.

The current exploratory study leverages transfer learning and open-access paediatric MRI datasets to improve the training procedure for this specific and novel task. We hypothesize that these techniques will enable accurate segmentation of paediatric brain tumours using DWI alone.

## Materials and methods

### Datasets

The primary dataset in the current retrospective study comprises data from paediatric brain tumour patients recruited from Birmingham Children’s Hospital for the Imaging of Tumors (IOT) Study, which is sponsored by the University of Birmingham, UK. Informed consent was given by parents/guardians for uploading patient data to the UK Children’s Cancer and Leukaemia Group Functional Imaging (CCLG-FIG) Database, including clinical variables and MRI data collected during their child’s clinical care and advanced imaging acquired under the research protocol. Ethical approval for the collection of this data and the current analyses was granted by regional and national research ethics committees (Reference: 04/MRE04/41). This dataset is hereafter referred to as the IOT cohort.

Additionally, this study utilizes data from the open-access MRI dataset obtained as part of the 2023 BraTS-PED^21,22^ project through Synapse ID (syn51156910). Secondary analysis of this data was granted ethical approval by the Aston University College of Health and Life Sciences Research Ethics Committee (#HLS21041).

### Patients

The IOT cohort included data from *n* = 107 paediatric brain tumour patients. Selection of the current cohort from the wider database was based on the following criteria:

- Patients with DWI data uploaded to the CCLG Database, at the diagnostic timepoint (i.e. typically before any adjuvant therapy/surgical intervention)
- Patients aged between 0 and 18 years

Cases were excluded if:

- DWI imaging contained significant and visible artefact (visually assessed by TM, JN and DJG-K)
- Reported radiological diagnosis was ‘diffuse’, thus presenting difficulties in accurately drawing manual delineations,
- Drawn ROI masks resulted in a too small tumour volume (see below)

Cases with missing MRI data were not included. Demographic characteristics of the patient cohort can be seen in Table 1.

**Table 1:** Demographics of included cohort

N	107
Sex (M:F)	54:53
Age (yrs)	
Mean	6.9
Range	0.0–16.3
Radio-clinical diagnosis	See Table S1

The BraTS-PED 2023 dataset is a retrospective cohort consisting of data from paediatric patients with high-grade glioma. Only data from the training (*n* = 99) cohort, where both the MRI and training labels/annotations are publicly available, was used in the current study.

### MRI data

DWI scans were acquired during standard care, at diagnosis, typically prior to surgical intervention or adjunct therapy, and thus are representative of the quality seen in clinical imaging studies. Imaging was conducted between 2005 and 2022. DWI was acquired across multiple scanners within the hospital setting (Table 2). The BraTS-PEDs dataset contains mpMRI sequences; T1w, T1w-CE, T2w and T2-FLAIR with details published elsewhere.^21^

**Table 2:** Diffusion MRI protocol parameters included in the primary cohort

Vendor	Model	Field strength (T)	Flip angle	Slice thickness (mm, min–max)	Spacing between slices (mm, min–max)	TE (ms, min–max)	Patients (*n*)
Siemens	Avanto	1.5	90	4–5	4–6.5	89–99	55
GE	SIGNA HDx	1.5	90	5	5–6	72.6–86.6	20
Siemens	Symphony	1.5	90	4–5	5.2–6.5	161	13
Phillips	Achieva	3	90	4–5	5–6	87.5–92.2	13
Siemens	Aera	1.5	90	4	4.4–5.2	89	6

Note: TE = echo time.

### Region of interest drawing

ROIs were manually drawn directly on *b0* images, using 3D Slicer^40^ (ver. 5.2.1). Additional imaging modalities (T1w, T2w, T1w-CE, FLAIR and *b1000*) were used as necessary to ensure inclusion of solid regions of tumour only (oedema and large cystic regions were excluded). Where discrepancies existed between modalities, b0 boundaries were ultimately used. For an internal region to be defined as cystic, it had to contain a minimum of four contiguous pixels within a single axial slice, with a cystic appearance in terms of contrast—to avoid exclusion of small non-cystic regions resulting from noise and/or artefact. Additionally, tumours had to be visible across more than two slices, with a voxel count greater than 100 in at least one slice to ensure a sufficient size for learning and segmentation. ‘Ground truth’ masks were generated through consensus review meetings. ROIs were initially drafted by TM, followed by iterative review/evaluation of these initial masks by JN and DJG-K and revision and editing of final masks by TM, until consensus was met. JN is a medical imaging scientist with 13 years of expert knowledge in paediatric MRI research, with significant experience delineating tumour tissue on MRI. DJG-K has 7 years of research experience in paediatric neuroimaging, including assessment of and drawing lesions for paediatric traumatic brain injury, demyelinating disease and brain tumours.

### Image preprocessing

DWI image sets were separated into constituent *b0* and *b1000* images. These images were bias-field (using the N4 algorithm from ANTs, v2.5.0^41^) and Gibbs artefact corrected (using the 2D degibbs algorithm^42^ as implemented in MRTrix v3.0.3^43^). Subsequently, patient-level images were resampled to 1 mm isotropic (using FLIRT^44,45^from the FSL package v6.0.7.6^46^), and voxel-wise image intensity was z-score normalized. The manually annotated ROI was also resampled to 1 mm isotropic.

From the raw b0 and b1000 signal, quantitative maps of ADC were calculated on a voxel-wise basis as^47^:


(1) $$\begin{array}{c} ADC=\frac{-\ln\;(S_{b}/S_{0})}{b} \end{array}$$

where *S_b_* is the observed signal intensity with field strength *b* (*in this case* b1000) and *S*_0_ is the observed b0 signal. ADC values were truncated between 0 and 4 *×* 10*^−^*^3^ mm^2^*/*s, as is common practice.^48,49^ Spatial maps of ADC were then resampled to 1 mm isotropic and intensity normalized. ADC maps have demonstrated good diagnostic utility but have also been shown to have good reproducibility across different scanners and acquisition parameters.^16^ The three pre-processed image types (b0, b1000, ADC), derived from the DWI, are the inputs for the models in the current study.

Details of the processing of the BraTS-PEDs 2023 dataset (beyond that performed by the data providers prior to sharing of the data) are described in the Supplementary material.

### Automated segmentation tool

The deep learning tool trained for the automated segmentation of tumours was DeepMedic (ver. 0.8.4).^35^ As outlined previously, DeepMedic is an 11-layer, multi-scale 3D CNN comprising normal- and low-resolution pathways, with the final three layers being fully connected. Outputs from the segmentation tool are probability maps representing the predicted probability of a voxel being classified as tumour versus non-tumour.

### Training and evaluation

All experiments were evaluated using 4-fold CV, with each fold consisting of training (*n* = 80/81) and testing data (*n* = 27/26), ensuring that partitions were disjoint at the patient level. Cases were randomly assigned to the folds, with the folds remaining constant/fixed over all presented experiments. All hyperparameters and model choices were based on model performance when inference was conducted on the training data (averaged across the four folds), whilst the final reported performance is evaluated based on inference conducted on testing cases, across all four folds. Two measures of performance are considered when evaluating the presented models and for optimizing parameters: Dice score coefficient and ‘failure’ rate.

Dice similarity coefficient (DSC),^50^ termed here as the Dice score, was used to quantify similarity between two sets of data, specifically the spatial concordance between the area labelled as tumour by the automatic segmentation and that labelled by the manual annotation (Dice = 0 being no elements in common, Dice = 1 being a perfect overlap). It is calculated by finding the intersection of the number of voxels labelled identically in both the manual and automated segmentations (labelled here as A and B), and the summed size (in voxels) of the underlying labels according to the following formula:


(2) $$\begin{array}{c} Dice=\frac{2\;|A\cap B|}{\left|A|+|B\right|} \end{array}$$

The predicted tumour masks are in the same resolution as the pre-processed input data: 1 mm isotropic. For evaluating segmentation quality, all predicted lesion masks were downsampled, returning to the resolution of the raw DWI scan (case-by-case), to account for the thick slice acquisition and slice gap. The Dice score is reported based on the congruence between this downsampled predicted tumour mask and the original/raw manually delineated mask. Together, these ensure applicability to downstream tasks utilizing quantitative imaging biomarkers extracted from the tumour ROI. ‘Failure’ rate was the number of individual cases/patients where the Dice score equals zero. That is to say that either the segmentation algorithm did not draw any predicted tumour region or the predicted mask did not overlap at all with the manual annotation. These were treated as ‘failed’ segmentations.

For the best-performing model (assessed using Dice and ‘failure’ rate), we also report Hausdorff distance 95% (HD95), positive predictive value (PPV), sensitivity and specificity—descriptions of which are provided in Table 3.

**Table 3:** Metrics for assessing the best-performing model

Metric	Description of metric
HD95	Measures the distance of the edges of the predicted ROI from the ground truth ROI, specifically in worst 5% of errors
PPV	Measures how many voxels in the predicted ROI are correct
Sensitivity	Measures how many of the voxels contained within the ground truth ROI are captured by the predicted ROI
Specificity	Measures how well voxels outside of the ground truth ROI are avoided by the predicted ROI

### Models and analysis

**Figure 1:**
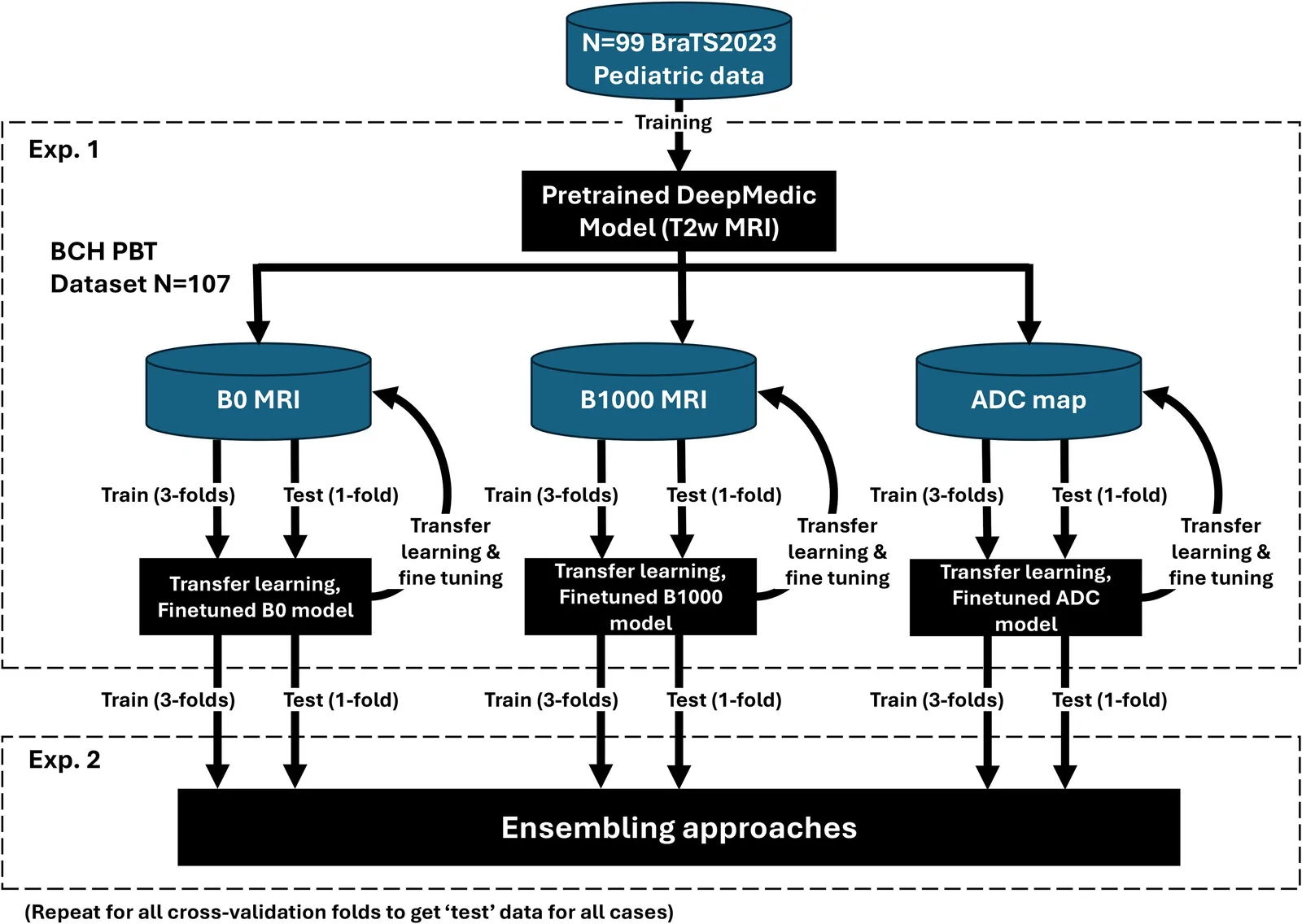
**Graphical outline of data workflow for given experiments/models.** This figure represents a single fold of the overall pipeline; this is repeated for all CV folds to assess test-data hold-out performance for all cases. N.B. BCH PBT = Birmingham Children’s Hospital Pediatric Brain Tumour, BraTS2023 = 2023 Brain Tumor Segmentation Pediatrics Tumor Challenge, ADC = apparent diffusion coefficient.

Figure 1 provides a visual overview of the analysis pipeline and associated workflow for the experiments outlined below.

#### Model 0: baseline models

To facilitate comparison of our adaptations to the default DeepMedic framework (outlined in Models 2 and 3), initial models were trained using only the IOT cohort (i.e. not using pre-training and trained from scratch). Training followed the same cross-fold approach as the other models. Three models were trained, one for each of the three image types (b0/b1000/ADC) as input, with the manual ROI annotation as the target label.

#### Model 1: transfer learning and fine-tuning

To account for the rarity of paediatric brain tumours, and the relatively low abundance of DWI data, which can affect the ability to train robust learners, we exploit benefits of transfer learning. An initial model was trained using data from the BraTS-PEDs dataset. As the dataset does not include DWI, to enable transfer learning whilst minimizing domain shift, this model was trained using T2w MRI as the input modality and optimized via manual tuning. This model is hereafter referred to as the BraTS-PEDs-T2w model.

Transfer learning to the target task was a two-step process. Firstly, the BraTS-PEDs-T2w model was used to initialize training on the IOT cohort as the target dataset. In this initial training, network weights in the initial layers were frozen, whilst the final layers were retrained. Then, all layers of the network were unfrozen, and all weights were updated using a learning rate an order of magnitude (×10) smaller than the initial learning rate used for pre-training. The number of final layers frozen (1, 2 or 3 from the final layer backwards) and the initial learning rate (2 × 10^−4^, 5 × 10^−4^, 1 × 10^−3^), following a polynomial schedule, were determined through grid search (3 × 3 grid space, inclusive of fine-tuning). Transfer learning from the BraTS-PEDs-T2w model to the IOT cohort was conducted for all three of the DWI image types (b0/b1000/ADC) independently.

#### Model 2: ensembling multiple inputs

Given the different imaging and contrast characteristics of each input image type (b0/b1000/ADC), it is likely that each model may perform differently, yet in a complementary manner, for the purposes of automated segmentation. Thus, to leverage the benefits of all three image types, we conducted ensembling of the output segmentations. Multiple ensembling methods were applied to combine the predictions of the models to assess the optimal approach (see Table 4).

**Table 4:** Methods of ensembling tumor predictions

Ensembling method	Description of method
Binary voting	Any voxel labelled as tumour by two of the three input models (i.e. a majority) is labelled as tumour.
Union mask	Any voxel labelled as tumour by any of the three input models (i.e. consensus) is labelled as tumour.
Intersection mask	Any voxel labelled as tumour by all three of the input models (i.e. consensus) is labelled as tumour.
Weighted voting	Voxel-wise average of tumour probability is calculated, and the resultant mask is then thresholded at DeepMedic’s default threshold of 0.5.

### Post-processing

Post-processing to improve the quality of segmentations involved both probability thresholding and erosion/dilation. Where the outputs of a model were probabilistic (i.e. every model other than the ensemble, which operates on binary outputs), DeepMedic’s default probability threshold for assigning a voxel as tumour versus background is 0.5. Using performance on the training data, we systematically tested a narrow window of probability thresholds (0.4, 0.5 and 0.7 selected based on prior experience with the tool) to optimize this arbitrary value. Once thresholded, the mask was then binarized for evaluation.

The low signal-to-noise ratio (SNR) of DWI (relative to other MRI modalities) can result in small, noisy components (likely false positives) in predicted tumour masks, which can be removed through post-processing approaches.^51^ Whilst previous studies and the BraTS-Ped challenge have used an area/volume threshold approach to deal with noisy segmentations,^20,51^ we refined the predicted tumour masks using a 2D convolutional erosion and dilation approach

For every predicted tumour mask, a 3 × 3, all-ones kernel was systematically passed over the whole image (within-slice) to erode (reduce in size) and/or dilate (increase in size) the mask, using OpenCV (v4.8.1). For each ROI, erosion was first applied and then dilation, up to three consecutive times for each type of manipulation. The purpose of this was to erode the mask, potentially removing small false positives, especially those distant from the actual tumour, and then expand the mask back out. Using the kernel/convolution approach means that these geometric distortions approximately retain the overall geometry of the predictions (whilst potentially introducing some error).

### Statistical analysis

In measuring the performance of our models, we report both mean and median Dice, as well as SD. We do not report statistical differences (e.g. *t*-tests) due to the large number of comparisons made (e.g. between optimization runs or between model comparisons, etc.) and therefore the risk of false positives. For the importance of benchmarking, we instead use the reported descriptive statistics, with absolute differences in Dice as a proxy for the magnitude of improvement between models.

However, statistical analyses were performed to assess potential biases in performance. Dice scores from the best-performing model (assessed in the test fold) were compared against clinical factors (tumour type and grade), patient factors (age and sex) and scan factors (MRI scanner model and field strength). Comparisons were conducted using *t*-test, one-way ANOVA and Spearman’s rank correlations. Where *post hoc t*-tests were used to compare across levels of the factor (e.g. grade) and therefore multiple comparisons were conducted, we used Bonferroni-corrected critical α values for assessing statistical significance within the tests for that confound. It should be noted that multiple comparison correction was only used within factors.

### Computation

The computations described in this paper were performed using two hardware configurations. Firstly, pre-training of the BraTS-PEDs-T2w model was conducted locally on a machine using two NVIDIA Quadro RTX 6000 (24 GB) GPUs and an AMD Threadripper Pro 3955WX CPU (16 cores, 32 threads). Training of all other DeepMedic models that used the IOT dataset was conducted using the University of Birmingham’s BlueBEAR HPC service, which provides a high-performance computing service to the university’s research community (http://www.birmingham.ac.uk/bear), specifically using NVIDIA Ampere A100 (40/80 GB) GPUs. Specific code developed in the current study (beyond tools described here and above) is available at https://github.com/tmulvanyAC/DWI_DeepMedic.

Visualizations of results were generated using ‘ggplot2’ (v3.5.1),^52^ ‘ggcorrplot’ (v0.1.4.1)^53^ and ‘ggExtra’ (v0.10.1),^54^ using R (v3.3.2)^55^ in RStudio (v2023.06.1.524).^56^

## Results

**Figure 2:**
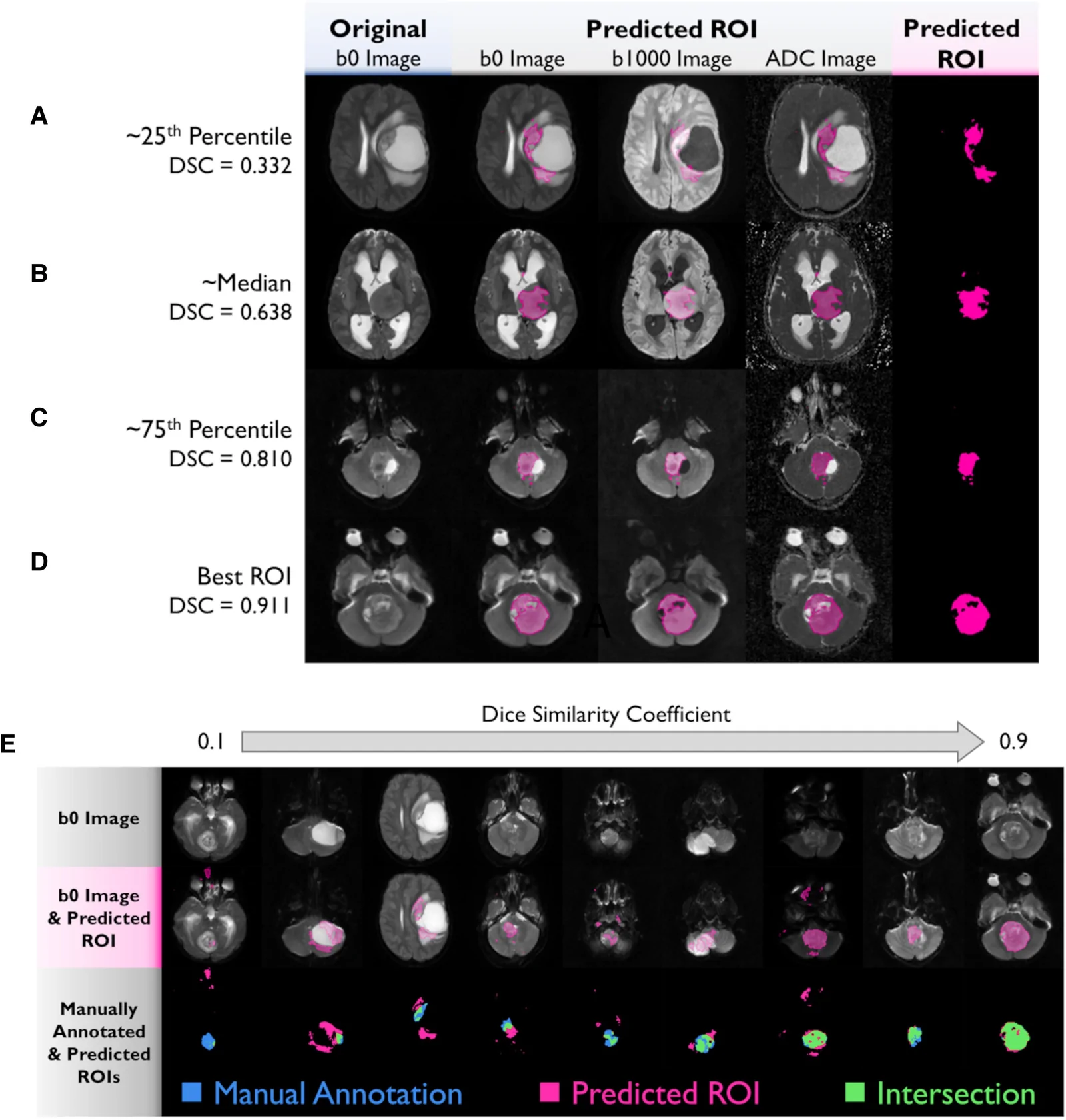
**Example automatic segmentations of tumours.** (**A–D**) Example predictions of increasing quality (from top row to bottom row), indicating performance on test fold, using the best-performing model (union-mask ensembled model). Rows represent individual patients selected based on their respective test-fold DSC, specifically selecting cases at given percentiles across the cohort: (**A**) 25th percentile (pilocytic astrocytoma patient); (**B**) 50th percentile, aka median (pilocytic astrocytoma patient); (**C**) 75th percentile (glioblastoma patient); and (**D**) best-performing case (medulloblastoma patient). Columns are (i) the b0 input image, (ii–iv) the predicted segmentation or ‘ROI’ overlaid upon the b0, b1000 and ADC input images (respectively) and (v) the predicted ROI. (**E**) To visually demonstrate the relationship between predicted segmentation and Dice score, a further selection of cases is displayed here. Cases were selected based on patient-level DSC; a case was sampled from each decile (from DSC = 0.1–0.9 in steps of 0.1) with performance closest to the desired DSC. The MRI slice visualized was selected based upon slice-level DSC, closest to the specific DSC decile. Cases have an increasing DSC from left to right. The top row shows the b0 image, the second row includes the predicted ROI overlaid on the b0 and the final row shows the intersection between the manual annotation and predicted ROI. This demonstrates the relative success of segmentations, even at mid-range deciles.

Results from the model evaluations can be seen in Table 5. Visualization of segmentations from the best-performing model can be seen in Fig. 2A and B.

**Table 5:** Performance of models on test data in terms of Dice score coefficient comparing predicted tumour masks to manual annotations

Model	Performance (test-fold)			
	Mean Dice	(SD)	Median Dice	Failure rate (cases with DSC = 0 (%))
Baseline models trained from scratch				
b0	0.36	0.32	0.33	2.8
b0 (+PP)	0.35	0.33	0.33	5.6
b1000	0.42	0.30	0.46	10.2
b1000 (+PP)	0.43	0.30	0.49	11.1
ADC	0.46	0.29	0.54	3.7
ADC (+PP)	0.45	0.29	0.51	7.4
Transfer learning and fine-tuning models				
b0	0.47	0.28	0.46	3.7
b0 (+PP)	0.48	0.28	0.50	7.4
b1000	0.41	0.28	0.44	13.0
b1000 (+PP)	0.43	0.28	0.48	13.9
ADC	0.43	0.29	0.49	5.6
ADC (+PP)	NA	NA	NA	NA
Ensemble of transfer-learning models (b0 + b1000 + ADC)				
Binary voting	0.51	0.29	0.59	5.6
Intersection mask	0.29	0.28	0.22	20.4
Union mask	0.55	0.28	0.63	8.3
Weighted voting	0.51	0.30	0.60	6.5

Note: Dice = Dice score coefficient, SD = standard deviation, PP = post-processing, ADC = apparent diffusion coefficient.

### Model 0

Of the baseline models, the ADC model performed most successfully, as assessed using Dice score ($\bar{Dice}(SD)=0.46(0.29)$), outperforming the b1000 model ($\bar{Dice}(SD)=0.42(0.30)$) and the b0 model ($\bar{Dice}(SD)=0.36(0.32)$). This result was echoed when assessed with median Dice for the cohort.

### Model 1

Transfer learning had the largest impact on b0 models (transfer learning $\bar{Dice}=0.47$ versus baseline$\bar{Dice}=0.36$), negligible impact on b1000 models (transfer learning $\bar{Dice}=0.41$ versus baseline $\bar{Dice}=0.42$) and reduced performance on the ADC model (transfer learning $\bar{Dice}=0.43$ versus baseline $\bar{Dice}=0.46$).

### Model 2

Of the tested ensembling methods, binary voting, union mask and weighted voting all improved the performance of the segmentation. Of these, the union mask approach performed most successfully ($\bar{Dice}(SD)=0.55(0.28)$), outperforming the next best single input model (b0 model with post-processing) by 0.07 whilst maintaining a similar SD across the cohort and rate of failed segmentations. Alternatively, the intersection mask approach significantly impacted performance ($\bar{Dice}(SD)=0.29(0.28)$), potentially due to the large number (>20%) of cases with ‘failed’ (Dice = 0) segmentations.

### ‘Failed’ segmentations

Across all trained models, the number of cases that failed segmentation (i.e. had a Dice score of 0) ranged from 2.8 to 13.9% for individual input models and from 5.6 to 20.4% in the ensembled models. Qualitatively considering this metric across models, a greater number of cases failed when b1000 images were used as inputs compared to b0 and ADC models.

When considering cases that performed with a Dice score of less than 0.05 across all models, 18 cases appeared in this bottom-performing group in five or more models (out of the 16 models described in Table 5). This perhaps suggests that these are ‘difficult’ cases that performed poorly irrespective of the model. Seven cases appeared in the bottom-performing group for nine or more models. Qualitative review of these cases did not indicate any patterns in terms of MRI scanner, field strength, age or sex. However, in terms of diagnosis, these cases included unbiopsied secreting germ cell tumour, glioblastoma, pilocytic astrocytoma, subependymal giant cell astrocytoma, germinoma, immature teratoma and unbiopsied optic pathway glioma. Notwithstanding the pilocytic astrocytoma, these are tumour types with fewer examples within the dataset for training. It is important to note that other tumour types with few cases do not appear in this list (e.g. atypical teratoid/rhabdoid tumor). However, 28 cases appeared in this bottom-performing group in only one or two models. This may suggest that the poor performance across models is, to some degree, unstable.

### Exploratory analysis

We performed analyses to compare patient-level Dice in relation to clinical, patient and scan-related factors. Dice scores from the best-performing model (union-mask ensembling of transfer-learned models: ADC + b0 + b1000) were compared against these factors. These results are visualized in Supplemental materials (Fig. S1). In terms of patient factors, no difference in Dice was identified due to sex (*P* > 0.05), nor was a significant correlation identified with age (*P* > 0.05). For scan factors, both magnet strength and scanner model seemed to affect Dice but, when taken together, seem to indicate that 3 T scans are performing worse than those at 1.5 T.

**Figure 3:**
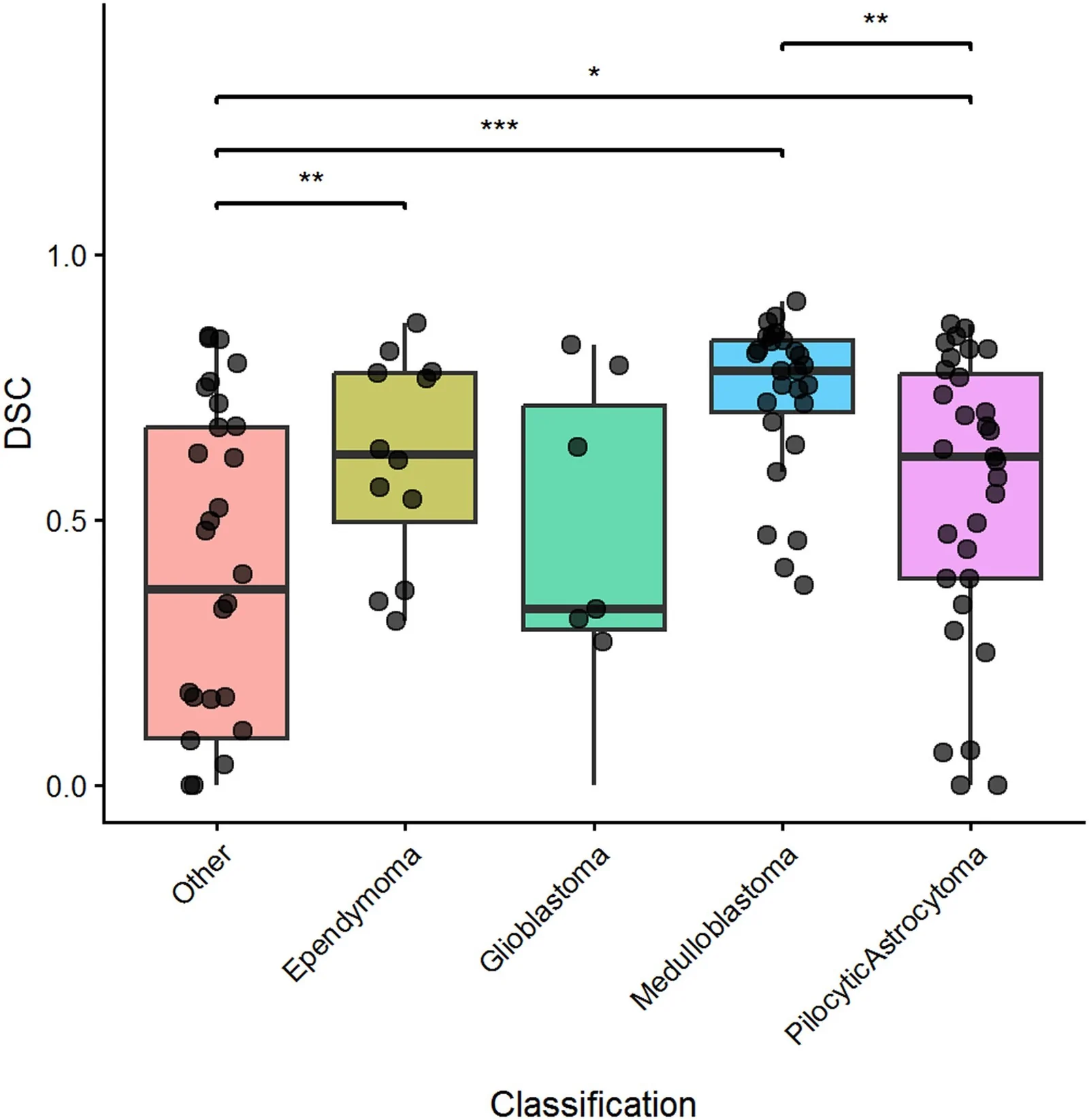
**Differences in Dice scores across different diagnostic classifications.** Dice was extracted from the best-performing model: union-mask ensembled model. Classifications represent a gross categorization notwithstanding histology, molecular or genetic subtyping of individual cases. Exploratory *t*-tests are conducted between factors, with significant differences reported (uncorrected for multiple comparisons) as per the following: *P < 0.05; **P < 0.01; ***P < 0.005. Given the multiple comparisons between diagnostic groups, only the difference between diagnoses of medulloblastoma and ‘other’ survived multiple comparison correction with a Bonferroni-corrected critical α of *P* < 0.005 (ependymoma *n* = 12, glioblastoma *n* = 7, medulloblastoma *n* = 27, pilocytic astrocytoma *n* = 31, other *n* = 30). Each datapoint represents an independent measure from a single patient.

In terms of clinical factors, no differences in terms of tumour grade (where labels were available) existed (*P* > 0.05), although significant differences existed between cases with no grading, versus grade 4 tumours—but this did not survive multiple comparison correction. Figure 3 also shows that when looking at a coarse classification of tumour type, segmentation of medulloblastoma seemed to significantly outperform other tumours when corrected for multiple comparisons (Fig. 3).

We also performed cross-correlations (Spearman’s-rank correlations) between patient-level Dice scores. Correlation matrices are displayed in the Supplementary material (Fig. S2). High rank correlations were found between many of the different models, suggesting that, overall, performance at the individual case level is relatively stable (i.e. cases that are performing well perform well regardless of the model, relative to the mean performance of the model). When comparing the rank correlations between baseline models and their transfer-learned counterparts, ADC and b1000 performance seemed stable, but there was a much lower rank correlation for the b0 model. Given that the mean Dice measure showed a large performance increase for the b0 model when transfer learning was applied, the lower rank correlation shows that the performance increase was equally distributed across individual cases; differences in performance rank must have come from some cases getting a larger increase in their performance than others. This is also borne out by the increase in median Dice for the cohort when transfer learning was applied.

To provide further evaluation of the best-performing model, HD95, PPV, sensitivity and specificity are reported in Table 6.

**Table 6:** Performance metrics for the best-performing model (union-mask ensembling of transfer-learned models: ADC + b0 + b1000)

Metric	Performance (test-fold)			
	Mean	Median	Minimum	Maximum
HD95	16.6	8.2	1	95.2
PPV	0.55	0.63	0	0.93
Sensitivity	0.66	0.74	0	0.98
Specificity	1.00	1.00	0.97	1.00

Note: HD95 = Hausdorff distance 95%, PPV = positive predictive value.

## Discussion

The current study proposes a deep-learning, CNN-based approach for automatically segmenting paediatric brain tumours from DWI. Overall, whilst qualitative review of predicted ROIs shows reasonable estimations of tumour, when assessed on Dice alone, performance was poorer than in similar benchmarking challenges (e.g. the 2023 BraTS-PEDs challenge, where the winning Dice score for tumour core was 0.80 using typical MR modalities^22^). Previous studies have incorporated DWI into deep-learning models for automated segmentation, alongside ‘standard’ imaging modalities (T1w, T2w, T1w contrast-enhanced and T2-FLAIR). They noted an additional benefit to segmentation accuracy when including the functional imaging data from DWI.^57,58^ These modest increases in performance may underestimate the independent value of DWI in a segmentation task, compared to our results. The current study demonstrates the ability to segment a diverse array of paediatric brain tumour types from DWI using CNN approaches.

It is important to note that the poorer performance here, compared to the reported performance of previous segmentation models, may be due to our evaluation approach. In the current study, we downsampled the predicted tumour ROIs output by DeepMedic, back into the geometry of the raw DWI data, before evaluating the segmentation. This was because the purpose of these tumour ROIs is to form the basis of an automated pipeline for conducting quantitative image analysis of the DWI scans (by extracting metrics, such as ADC, from the ROI). It is therefore important that the model segmentation performs well relative to the native DWI image space, rather than resampling and interpolating the DWI signal. The fact that the original data is interpolated for these purposes, it is possible that artefacts could diminish segmentation performance, and our resampling approach aims to address this. Nonetheless, previous research conducting segmentations of brain anatomy has suggested robustness of Dice to downsampling, up to 50% of the original resolution.^59^ This previous study resampled to an arbitrary resolution, whereas we downsampled to match the resolution of the original input MRI, corresponding to around 15–25% downsampling (in a single plane), which is well within the limits of the 50% limit discussed here.

The current results demonstrate the added value of cross-modal transfer learning for this task, pre-training the model on T2w MRI and fine-tuning on our DWI data. This is a vital step in this work given that DWI is not well represented in available public tumour datasets, severely limiting opportunities to increase training dataset sizes. Unsurprisingly, the benefit of this cross-modal approach was only borne out of the b0 model, likely due to the much smaller domain shift—MR contrast between b0 and T2w MRI is much more similar than between T2w and b1000 (where SNR is also lower) or ADC (where the data being represented is fundamentally different). This represents a potential bias towards the b0 inputs in our model. These results suggest that further iterations of the model should avoid transfer learning for the ADC images, in favour of training from scratch. However, this will remain challenging under current sample sizes (one of the major problems we tried to address with transfer learning). It is hoped that in the future, DWI will be better represented in large, consortia-led open MRI datasets in paediatric brain tumours—thus eliminating the need for cross-modal transfer learning across any of the imaging inputs (b0, b1000 or ADC), where domain shift is likely to curtail an added benefit. Efforts towards larger open-access datasets will eventually remove the need for transfer learning altogether.^38^ However, given current data availability, especially in the case of DWI, our results indicate that this remains a viable solution to improving model training.

Whilst we attempted to reduce domain shift between modalities for transfer learning, there were other differences. The BraTS cohort, on which these models were pre-trained,^21^ is relatively homogeneous in terms of tumour types, typically being high-grade gliomas (e.g. high-grade astrocytoma, diffuse midline glioma (DMG) and diffuse intrinsic pontine glioma (DIPG)). This results in significant differences between the population being trained on and our patient cohort, even across features, such as tumour volume and location. In terms of location, the patch-based approach of DeepMedic should be robust. However, in terms of tumour size, the optimal patch size for the CNN is related to tumour volume,^60^ and this may therefore have impacted our results. Related biases may occur due to the relative sensitivity of DWI, specifically ADC, to different tumour types. For instance, in paediatrics, medulloblastomas show greater restricted diffusion (lower mean ADC values) than pilocytic astrocytomas.^3^ Due to the rarity of paediatric brain tumours and the limited available data for transfer learning, these challenges may be unavoidable; however, a semi-automated approach, selecting a cohort/model with the most appropriate transfer learning for the target cohort you wish to segment (e.g. based on location, tumour type or adults versus children), may hold value for future clinical implementation.

Similar to previous studies, our segmentation approach displayed long tails in terms of Dice score metrics; whilst many cases performed well, some performed poorly, including cases with Dice scores of zero. A previous study segmenting tumour subregions from mpMRI using DeepMedic had long tails, demonstrating Dice scores of zero.^19^ A previous study conducting automatic segmentation of paediatric low-grade glioma from T2w MRI (using a similar transfer learning approach to the current study) found that, across models, 6–15% of cases had a Dice score of zero.^38^ This suggests that generalizability of automated segmentation models still remains a challenge, and that this is not exclusive to the model setup of the current study. Potential mitigations may be developed in the future through understanding the types of cases where the model performs poorly (through thorough failure analysis, such as that in the current study), allowing clinicians in a clinical workflow to select and refer those cases that are most challenging for the tool to be managed manually. This will ensure that, where time-consuming manual segmentation needs to be used, it is only used for cases that we know will require it, thereby allowing this limited source to be directed more efficiently.

Whilst we outline multiple metrics as the standard for evaluating these models, the overall goal of such an approach is to achieve clinical translation and deployment. There is no current research to support the threshold at which automated segmentations are ‘good enough’ for the purposes of clinical tasks. There is a clear need for the assessment of the accuracy of segmentation at the individual patient level and how that influences tasks, such as the reliable extraction of ADC biomarkers. However, there is also a need for heuristics to understand whether the performance of any given model [as measured with cohort-level mean (SD) Dice] is sufficient for performing clinical tasks. In the current study, the model is likely insufficient for this task, not because of the poorer cohort-level Dice score, but instead due to the failure rates seen in the current models. Overall, this highlights that reporting of Dice alone for this and future models is unlikely to inform the specific usefulness of these models for these clinical tasks. Therefore, future work will need to move away from reporting Dice and instead focus on the downstream impacts of automated versus manual segmentations, for tasks such as diagnostic classification, for instance (similar to^7^).

### Strengths and limitations

A significant strength of the current study is the use of paediatric data for pre-training and transfer learning, whereas other studies have pre-trained on adult data alone. Previous studies with access to both adult and paediatric brain tumour cohorts seemingly suggest that the task of paediatric brain tumour segmentation is potentially harder than that in adult cohorts. Cohort-level segmentation performance has been shown to be poorer for paediatric versus adult cohorts, even when using the same deep-learning architectures.^18^ Across medical imaging segmentation tasks, deep-learning models trained on adult data do not perform well when applied to paediatric cases,^38,61^including in the context of brain tumour segmentation.^38,62^ It is currently unclear whether this is due to reduced sample sizes in paediatrics for model training, or due to the phenotypic differences that exist between paediatric and adult tumours.^63,64^ This may explain why recent research has demonstrated that skull-stripping algorithms trained on adult data generalize well when applied to paediatric data^62^—the radiological phenotype of the skull does not differ greatly between these two age groups. However, this clearly highlights the greater need for paediatric-focused approaches, rather than assuming that adult approaches will be valid for this population.

The current study utilized an adapted DeepMedic framework. We specifically chose DeepMedic for the support and implementation of transfer learning, a key aspect of the training framework presented here. However, many recent developments in research aiming to segment brain tumours have utilized U-Net algorithms,^65^ including architectures, such as nnU-Net. nnU-Net is an adaptive, out-of-the-box, deep-learning segmentation approach designed for specific task-optimization.^66,67^ nnU-Net featured heavily in top-performing models in the BraTS-PEDs 2023 challenge (a challenge aimed at the segmentation of paediatric brain tumours using MRI)^22^ and in previous adult segmentation challenges.^67,68^ However, recent studies conducting brain tumour segmentation in paediatrics have shown mixed results regarding the supposed superiority of nnU-Net over DeepMedic. When applied to a paediatric cohort, the default configuration of DeepMedic outperformed nnU-Net when segmentations of tumour subregions were evaluated using HD95.^18^ Alternatively, a similar study that specifically compared DeepMedic and nnU-Net showed significantly better performance for paediatric tumour segmentation when using nnU-Net.^19^ It is important to note, given the long-term goal of clinical implementation of any such work, that DeepMedic has lower computational demands than nnU-Net. There is also extremely limited support for transfer learning in the current nnU-Net implementation. Future approaches to DWI segmentation may employ the nnU-Net architecture instead of DeepMedic and should compare the relative performance of each.

One potential limitation is the single-channel input of our model and subsequent late fusion of predictions via ensembling, rather than using a multi-channel model with input-level fusion.^69^ This was an inherent limitation of our model setup, conducting cross-modal transfer learning from the same one input modality (T2w) to each of the target modalities (b0, b1000 and ADC, respectively) required us to train each diffusion input as a separate model, rather than doing early fusion through a multi-input model. There is no purported evidence to suggest whether prediction- or input-level fusion benefits the task of tumour segmentation, but input-level fusion may allow the model to learn a fused feature representation across ‘modalities’ (in this case across b0, b1000 and ADC images), potentially improving segmentation outcomes. However, the current study did not employ this approach due to the single-modality transfer learning employed—thus these fused feature representations could not be pre-learnt and fine-tuned in our current dataset. With a larger dataset, it could be directly tested as to whether the fine-tuning approach evaluated in the current study over- or underperforms compared to this multi-modal fusion, to determine which approach holds more value in this task.

Another potential limitation is that we conducted no correction to account for EPI (echo-planar imaging) geometric distortion in the DWI. In our results, we found that 1.5T data outperformed 3T data. This may be because EPI distortion is greater at higher MRI field strengths, thus limiting the performance of our segmentation model. However, limited evidence suggests that EPI distortion correction (both at the level of acquisition and post-processing) does not significantly change the performance of an automated segmenter using DWI as an additional input modality.^70^ However, this lack of difference may be because DWI only represented one-fifth of all input modalities in this study. Also, in a typical DWI acquisition, geometric distortions are focused in the phase-encoding direction, with the anterior frontal regions experiencing the greatest artefact and so the benefit of correcting this artefact may be limited only to those cases with tumours in this location (a minority of paediatric brain tumours)—not providing benefit to segmentation performance at the cohort level. Previous studies using DWI as the sole input for deep-learning and automated segmentation also did not correct for this.^25-28^

## Conclusion

Delineation of whole tumour volumes is vital to further develop quantitative medical image analysis of paediatric brain tumours, using functional imaging modalities not limited to DWI. However, as international collaborations increase, and datasets reach the larger sample sizes necessary to leverage the benefits of machine learning, manual delineation will be a challenging bottleneck preventing the clinical impact of this research from being delivered. This project provides a promising proof-of-concept for how automated segmentation of paediatric brain tumours from DWI may help solve this bottleneck and support clinical translation into tools, such as an automated *in vivo* diagnostic classifier.

Future work is needed to establish the downstream impacts of the proposed automated pipeline (for instance, on volumetric measurements or ADC feature extraction), to use health-economic analysis to establish the clinical-value proposition and finally to provide the necessary regulatory approvals to support clinical deployment.

## Supplementary Material

fcag317_Supplementary_Data

## Data Availability

Due to the sensitive nature of the data used in this study, which involves child patient data, the data cannot be shared publicly. Access to the data is restricted in accordance with ethical guidelines and study protocol. Specific code developed in the current study (beyond tools described here and above) is available at https://github.com/tmulvanyAC/DWI_DeepMedic.
