# Supplementary material for: Deep learning segmentation of paediatric brain tumours using diffusion-weighted MRI: towards an early, in vivo classification pipeline
Source: Brain Commun. 2026 Aug 17;8(4):fcag317. doi: 10.1093/braincomms/fcag317 (PMC13504704; doi:10.1093/braincomms/fcag317)
Supplement: Supplementary file 1 [file fcag317_supplementary_data.docx]

Deep learning segmentation of pediatric brain tumors using diffusion-weighted MRI: towards an early, in-vivo classification pipeline

Daniel Griffiths-King, Timothy Mulvany, Heather Rose, Andrew Peet, John Apps, Theodoros N Arvanitis, & Jan Novak

**Supplementary Materials**

1. **Preprocessing of BraTS-PEDs 2023 dataset**

The BraTS-PEDs 2023 dataset was preprocessed prior to pre-training of the BraTS-PEDs-T2w model, with processing similar to that of the CCLG-FIG dataset. Whilst the dataset contains multiparametric / multimodal MRI data, the current study utilizes only the T2w MRI, with closest expected tissue contrast/weighting to the DWI. The publicly shared dataset is already pre-processed in accordance with description in the challenge manuscripts [1, 2], which briefly includes co-registration to a standard anatomical template space, resampling to 1mm isotropic, and defacing of images to remove potentially identifiable facial features (as part of the de-identification process). We conducted additional preprocessing locally on these images, including bias field correction, using the N4 algorithm (from ANTs [3]) and Gibbs artefact correction, using the 2D degibbs algorithm [4] as implemented in MRtrix ([5]). Because of the nature of the publicly available dataset already having some preprocessing conducted, this does in fact mean that the processes applied to the two datasets are similar, but not identical, especially in terms of order of when resampling occurred.

The BraTS-PEDs 2023 dataset also contains tumor annotations (which are semi-automated, with heavy manual intervention to revise and adapt predicted segmentations, see [1, 2] for details) which include tumor subregions, including enhancing tumor, non-enhancing tumor, cystic component and edema. For the purposes of our pretrained model, we combined the enhancing, non-enhancing and cystic regions to generate a ‘Tumor Core’ mask which was used as the target label for the BraTS-PEDs-T2w model.

| **Table S1.** Clinical descriptors of cases in the cohort | |
| --- | --- |
| **Radio-Clinical Diagnostic Classification** | **n** |
| Anaplastic Ependymoma | 6 |
| Anaplastic Astrocytoma | 2 |
| Anaplastic Medulloblastoma | 1 |
| Astroblastoma | 1 |
| Atypical Choroid Plexus Papilloma | 1 |
| Atypical Teratoid - Rhabdoid Tumour | 1 |
| CNS Primitive Neuroectodermal Tumour | 1 |
| Choroid Plexus Carcinoma | 2 |
| Choroid Plexus Papilloma | 1 |
| Desmoplastic-Nodular Medulloblastoma | 3 |
| Diffuse Midline Glioma, H3 K27M-mutant | 1 |
| Dysembryoplastic Neuroepithelial Tumour | 1 |
| Ependymoma | 6 |
| Ganglioglioma | 3 |
| Germinoma | 3 |
| Glioblastoma | 7 |
| Medulloblastoma | 23 |
| Mixed Germ Cell Tumours | 1 |
| Pilocytic Astrocytoma | 30 |
| Pilomyxoid Astrocytoma | 2 |
| Pineoblastoma | 1 |
| Pineocytoma | 1 |
| Subependymal Giant Cell Astrocytoma | 2 |
| Teratoma - Immature | 1 |
| Teratoma - Mature | 1 |
| Unbiopsied Secreting Germ Cell Tumour | 1 |
| Unbiopsied Optic Pathway Glioma | 4 |

**C)**

**D)**

**B)**

**A)**

**Figure S1.** Graphs indicating potential biases in Dice scores across the cohort, for the best performing model (Union Mask Ensemble Model). Biases are assessed across both scanning factors (A) Magnet/Field Strength, B) Scanner Model) and patient factors (C) Patient Sex, D) Tumour Grade). Exploratory T-tests (A + C), and one-way ANOVA with post-hoc t-tests are conducted to compare levels of each factor. Significant differences are reported (uncorrected for multiple comparisons) as per the following: **p* < 0.05, ***p* < 0.01. Only the comparison between magnetic field strength survived multiple comparison correction. Data points are individual patients in graphs where a subset of the data has a group size of n<10.

**C)**

**A)**

**B)**

**Figure S2.** Cross-correlation matrices indicating correlation (Rank-correlation using Spearman’s Rho, n=107) between Dice scores for each model, specifically A) Baseline, B) Transfer-learned and C) Ensembled models. High rank correlations suggest that overall, performance at the individual case level is relatively stable (i.e. cases that are performing well, perform well regardless of model, relative to the mean performance of the model), but performance ranking became highly stable once ensembling is used (C). Correlations between Baseline and transfer learned models for each modality (not displayed in above figure), showed that whilst the ADC and B1000 performance (in terms of Dice) was highly corelated, this was not the case for the B0 model (ADC Models: Rho(Baseline, Transfer)=0.91, B0 Models: Rho(Baseline, Transfer)=0.67, B1000 Models: Rho(Baseline, Transfer)=0.92) potentially indicating that the performance increase to the B0 model provided by Transfer learning was not a uniform increase in performance across cases, but some cases benefited more greatly than others from this methodology.

**References**

1.	Kazerooni, A.F., et al., *The Brain Tumor Segmentation (BraTS) Challenge 2023: Focus on Pediatrics (CBTN-CONNECT-DIPGR-ASNR-MICCAI BraTS-PEDs).* ArXiv, 2024.

2.	Kazerooni, A.F., et al., *BraTS-PEDs: Results of the Multi-Consortium International Pediatric Brain Tumor Segmentation Challenge 2023.* arXiv preprint arXiv:2407.08855, 2024.

3.	Tustison, N.J., et al., *N4ITK: improved N3 bias correction.* IEEE Trans Med Imaging, 2010. **29**(6): p. 1310-20.

4.	Kellner, E., et al., *Gibbs-ringing artifact removal based on local subvoxel-shifts.* Magn Reson Med, 2016. **76**(5): p. 1574-1581.

5.	Tournier, J.D., et al., *MRtrix3: A fast, flexible and open software framework for medical image processing and visualisation.* NeuroImage, 2019. **202**: p. 116137.
